# The effect of semaglutide on blood pressure in patients with type-2 diabetes: a systematic review and meta-analysis

**DOI:** 10.1007/s12020-023-03636-9

**Published:** 2023-12-15

**Authors:** Wei Wu, Huo-mu Tong, Yun-sheng Li, Jia Cui

**Affiliations:** 1 Department of Geriatrics, Chun’an First People’s Hospital (Zhejiang Provincial People’s Hospital Chun’an Branch), Hangzhou, 311700 Zhejiang China; 2 Department of Endocrinology, Chun’an First People’s Hospital (Zhejiang Provincial People’s Hospital Chun’an Branch), Hangzhou, 311700 Zhejiang China

**Keywords:** Semaglutide, Blood pressure, Type-2 diabetes, Meta-analysis

## Abstract

**Objective:**

To evaluate the blood pressure (BP) lowering ability of semaglutide, a glucagon-like peptide-1 receptor agonist (GLP-1 RA), in individuals with type-2 diabetes (T2D).

**Methods:**

Randomized controlled trials (RCTs) comparing subcutaneous or oral semaglutide with placebo or other antihyperglycemic agents (AHAs) in T2D patients were identified by searching PubMed, Embase, Web of Science, ClinicalTrials.gov and Cochrane Library. These screened studies included the outcomes of interest: systolic and/or diastolic BP. Weighted mean differences (WMDs) and 95 % confidence intervals (CIs) were used to present the meta-analysis results. Pooled and sensitivity analyses were performed, and the risk of bias was evaluated.

**Results:**

Twenty-nine RCTs with a total of 26985 participants were recruited in the final analysis. The WMD in change from baseline in systolic BP (SBP) of semaglutide versus placebo or other AHAs was −2.31 mmHg (95% CI: −3.11 to −1.51), while that for diastolic BP (DBP) was 0.09 mmHg (95% CI: −0.16 to 0.33). It also reduced glycated hemoglobin A1c (HbA1c) by 0.75% (95% CI: −0.92 to −0.58) and body weight loss by 2.80 kg (95% CI: −3.51 to −2.08). The reduction in SBP was similar for subcutaneous and oral administration of semaglutide, with −2.36 (95% CI: −3.38 to −1.35) and −2.50 (95% CI: −3.48 to −1.53), respectively.

**Conclusions:**

In T2D, SBP decreased significantly in the semaglutide group compared with placebo or other active controls. According to the efficacy results from this meta-analysis, subcutaneous and oral semaglutide have similar SBP-reducing effects. Therefore, the treatment of T2D patients with subcutaneous semaglutide or oral preparations is beneficial for reducing SBP.

## Introduction

Diabetes mellitus affected more than 537 million adults worldwide in 2021, with nearly 90% of cases being type-2 diabetes (T2D) [1]. Its prevalence is still rising steadily, which constitutes a substantial health burden to society. Hypertension occurs in two-thirds of individuals with diabetes and can lead to an increased risk of cardiovascular disease (CVD), chronic kidney disease (CKD), stroke, retinopathy, and even death [1]. Previous studies have shown that there is always poor control of blood pressure (BP) in patients with T2D. Therefore, optimal lowering of BP is important for the management of subjects with T2D [2]. If antihyperglycemic drugs can also reduce BP, there will be obvious benefits for T2D patients.

Semaglutide is a GLP-1 RA that has been developed for the treatment of T2D, and higher doses are approved to treat obesity. It is the only GLP-1 RA recently available as both once-daily oral and once-weekly subcutaneous [3]. Oral semaglutide was the first oral GLP-1 RA authorized by the Food and Drug Administration (FDA) for the treatment of T2D on September 20th, 2019 [4]. Compared with subcutaneous semaglutide, oral formulation has different absorption characteristics. However, the pharmacokinetic functions and properties of semaglutide after absorption are similar regardless of the route of administration [5].

Semaglutide exerts glucose-lowering effects in a glucose-dependent manner, thus not increasing the risk of hypoglycemia. Apart from lowering glycated hemoglobin A1c (HbA1c), semaglutide also reduces weight by suppressing gastric peristalsis and inhibiting appetite. In addition, it has been found to benefit subjects with T2D in terms of BP regulation, oxidative damage, albuminuria reduction, kidney function improvement, and more. In particular, the regulation of BP in recent years has attracted widespread concern. However, few studies have examined the effect of semaglutide on BP as a primary outcome. Therefore, this review analyzed the results of multiple RCTs to further investigate the effect of semaglutide on BP levels in individuals with T2D.

## Methods

### Search strategy

The review was conducted according to the 2020 PRISMA (Preferred Reporting Items for Systematic Reviews and Meta-Analysis) guidelines, which were registered under PROSPERO (CRD42023406008) [6, 7]. Web of Science, Embase, the Cochrane Library, PubMed and Clinicaltrials.gov were systematically searched from inception until March 18, 2023, to investigate the effect of semaglutide on BP in patients with T2D. Changes from baseline in SBP and/or DBP were the primary outcomes for the included trials. We combined “semaglutide,” “Rybelsus,” “Ozempic,” “Diabetes Mellitus, Type 2,” “Diabetes Mellitus, Noninsulin Dependent,” and “randomized controlled trial” as either the Medical Subject Headings (MeSH) terms or keywords. The results were further limited to human studies published in English.

### Inclusion criteria and exclusion criteria

Studies were included if (a) participants were 18 years of age or older with T2D; (b) they compared the effect of subcutaneous or oral semaglutide with other antihyperglycemic agents (AHAs) or placebo in regulating BP; (c) they reported interesting outcomes; and (d) they were RCTs. Studies were excluded if they were (a) nonhuman studies; (b) duplicate publications; (c) non-RCTs; (d) nonoriginal studies; (e) not reporting information of interest; or (f) non-English.

### Study selection and data extraction

First, W.W. used EndNote reference software to remove duplicate studies. Second, two authors (W.W., H.-M.T.) independently screened from each paper and extracted detailed data using standardized forms. Disagreements were resolved via consensus.

The demographics of the included studies included author, study, publication year, study duration, study arms, sample size, average age, sex ratio, diabetes duration, HbA1c and body weight (Table 1). Changes from baseline in systolic and/or diastolic BP were the primary outcome for the included trials. Additionally, we also extracted data on changes in HbA1c and body weight. In cases where the data for changes from baseline could not be obtained, suitable information for calculating changes from baseline was extracted.

**Table 1:** Demographic characteristics of included studies

Author	Study	Year	Study duration	Study arms	Sample size	Average age (years)	Female (%)	Diabetes duration	HbA1c (%)	Body weight (kg)
Marso et al. [10]	SUSTAIN 6	2016	104-week	Semaglutide 0.5 mg	826	64.6 ± 7.3	40.1	14.3 ± 8.2	8.7 ± 1.4	91.8 ± 20.3
				Semaglutide 1.0 mg	822	64.7 ± 7.1	37.0	14.1 ± 8.2	8.7 ± 1.5	92.9 ± 21.1
				Placebo 0.5 mg	824	64.8 ± 7.6	41.5	14.0 ± 8.5	8.7 ± 1.5	91.8 ± 20.3
				Placebo 1.0 mg	825	64.4 ± 7.5	38.5	13.2 ± 7.4	8.7 ± 1.5	91.9 ± 20.8
Sorli et al. [20]	SUSTAIN 1	2017	30-week	Semaglutide 0.5 mg	128	54.6 (11.1)	53	4.81 (6.10)	8.09 (0.89)	89.81 (22.96)
				Semaglutide 1.0 mg	130	52.7 (11.9)	38	3.62 (4.88)	8.12 (0.81)	96.87 (25.59)
				Placebo	129	53.9 (11.0)	46	4.06 (5.48)	7.95 (0.85)	89.05 (22.16)
Ahren et al. [33]	SUSTAIN 2	2017	56-week	Semaglutide 0.5 mg	409	54.8 (10.2)	49	6.4 (4.7)	8.0 (0.9)	89.9 (20.4)
				Semaglutide 1.0 mg	409	56.0 (9.4)	50	6.7 (5.6)	8.0 (0.9)	89.2 (20.7)
				Sitagliptin 100 mg	407	54.6 (10.4)	49	6.6 (5.1)	8.2 (0.9)	89.3 (19.7)
Aroda et al. [26]	SUSTAIN 4	2017	30-week	Semaglutide 0.5 mg	362	56.5 (10.3)	46	7.8 (5.1)	8.1 (0.8)	93.7 (21.4)
				Semaglutide 1.0 mg	360	56.7 (10.4)	49	9.3 (7.2)	8.3 (0.9)	93.7 (21.4)
				Insulin glargine	360	56.2 (10.6)	46	8.6 (6.3)	8.1 (0.9)	92.6 (21.5)
Davies et al. [32]		2017	26-week	Semaglutide 2.5 mg	70	56.7 (9.9)	35.7	6.1 (6.0)	8.0 (0.7)	93.6 (15.6)
				Semaglutide 5 mg	70	55.7 (11.0)	32.9	5.3 (4.7)	7.8 (0.6)	93.1 (19.0)
				Semaglutide 10 mg	69	56.5 (10.1)	37.7	5.8 (4.8)	7.8 (0.7)	91.8 (14.0)
				Semaglutide 20 mg	70	58.3 (10.4)	37.1	7.0 (5.3)	7.9 (0.7)	93.8 (17.9)
				Semaglutide 40 mg	71	56.5 (10.2)	39.4	7.7 (5.9)	8.0 (0.7)	90.8 (16.5)
				Semaglutide 40 mg (Slow Escalation)	70	57.1 (10.5)	41.4	6.6 (4.9)	8.0 (0.7)	93.3 (18.8)
				Semaglutide 40 mg (Fast Escalation)	70	57.7 (10.8)	37.1	5.6 (4.7)	7.8 (0.8)	92.0 (15.4)
				Semaglutide 1.0 mg	69	56.8 (11.8)	30.4	5.6 (5.0)	7.8 (0.7)	88.8 (15.4)
				Placebo	71	58.9 (10.3)	43.7	6.7 (5.1)	8.0 (0.8)	93.8 (18.1)
Ahmann et al. [36]	SUSTAIN 3	2018	56-week	Semaglutide 1.0 mg	404	56.4 (20–82)	45.8	9.0 (0.4–37.1)	8.4 (6.7–11.1)	96.2 (49.9–198.3)
				Exenatide ER2.0 mg	405	56.7 (21–83)	43.7	9.4 (0.3–54.0)	8.3 (6.5–11.2)	96.2 (49.9–198.3)
Rodbard et al. [17]	SUSTAIN 5	2018	30-week	Semaglutide 0.5 mg	132	59.1 (28–84)	56.1	12.9 (0.4–37.1)	8.4 (7.0–10.3)	92.7 (50.4–162.8)
				Semaglutide 1.0 mg	131	58.5 (33–80)	58.8	13.7 (0.6–36.9)	8.3 (6.9–10.8)	92.5 (48.5–165.6)
				Placebo	133	58.8 (19–86)	53.4	13.3 (0.8–39.6)	8.4 (6.8–11.1)	89.9 (47.5–157.3)
Pratley et al. [14]	SUSTAIN 7	2018	40-week	Semaglutide 0.5 mg	301	56 (10.9)	44	7.7 (5.9)	8.3 (0.9)	96.4 (24.4)
				Semaglutide 1.0 mg	300	55 (10.6)	46	7.3 (5.7)	8.2 (0.9)	95.5 (20.9)
				Dulaglutide 0.75 mg	299	55 (10.4)	46	7.0 (5.5)	8.2 (0.9)	95.6 (23.0)
				Dulaglutide 1.5 mg	299	56 (10.6)	43	7.6 (5.6)	8.2 (0.9)	93.4 (21.8)
Seino et al. [28]		2018	30-week	Semaglutide 0.5 mg	103	58.8 (10.4)	23.3	8.0 (5.2)	8.2 (1.0)	67.8 (11.7)
				Semaglutide 1.0 mg	102	58.1 (11.6)	26.5	7.8 (6.9)	8.0 (0.9)	70.8 (16.4)
				Sitagliptin 100 mg	103	57.9 (10.1)	21.4	8.1 (6.7)	8.2 (0.9)	69.4 (12.9)
Kaku et al. [29]		2018	56-week	Semaglutide 0.5 mg	239	58.0 (10.6)	30.5	8.1 (6.0)	8.0 (0.9)	71.0 (15.4)
				Semaglutide 1.0 mg	241	58.7 (10.2)	27.8	9.4 (6.5)	8.1 (1.0)	71.7 (15.9)
				Additional OAD	120	59.2 (10.1)	25.8	9.3 (7.0)	8.1 (0.9)	72.2 (14.9)
Aroda et al. [35]	PIONEER 1	2019	26-week	Semaglutide 3 mg	175	55 (11)	49.1	3.8 (5.3)	7.9 (0.7)	86.9 (21.0)
				Semaglutide 7 mg	175	56 (11)	46.9	3.6 (5.1)	8.0 (0.6)	89.0 (21.8)
				Semaglutide 14 mg	175	54 (11)	50.9	3.4 (4.4)	8.0 (0.7)	88.1 (22.1)
				Placebo	178	54 (11)	50.0	3.4 (4.6)	7.9 (0.7)	88.6 (23.4)
Rodbard et al. [37]	PIONEER 2	2019	52-week	Semaglutide 14 mg	411	57 (10)	49.9	7.2 (5.8)	8.1 (0.9)	91.9 (20.5)
				Empagliflozin 25 mg	410	58 (10)	49.0	7.7 (6.3)	8.1 (0.9)	91.3 (20.1)
Rosenstock et al. [13]	PIONEER 3	2019	78-week	Semaglutide 3 mg	466	58 (10.0)	45.5	8.4 (6.1)	8.3 (1.0)	91.6 (22.0)
				Semaglutide 7 mg	465	58 (10.0)	47.3	8.3 (5.8)	8.4 (1.0)	91.3 (20.8)
				Semaglutide 14 mg	465	57 (10.0)	46.9	8.7 (6.1)	8.3 (0.9)	91.2 (21.7)
				Sitagliptin 100 mg	467	58 (10.0)	49.0	8.8 (6.0)	8.3 (0.9)	90.9 (21.0)
Pratley et al. [16]	PIONEER 4	2019	52-week	Semaglutide 14 mg	285	56 (10)	48	7.8 (5.7)	8.0 (0.7)	92.9 (20.6)
				Liraglutide 1.8 mg	284	56 (10)	48	7.3 (5.3)	8.0 (0.7)	95.5 (21.9)
				Placebo	142	57 (10)	48	7.8 (5.5)	7.9 (0.7)	93.2 (20.0)
Mosenzon et al. [21]	PIONEER 5	2019	26-week	Semaglutide 14 mg	163	71 (8)	49	14.1 (8.6)	8.0 (0.7)	91.3 (17.8)
				Placebo	161	70 (8)	55	13.9 (7.4)	7.9 (0.7)	90.4 (17.5)
Husain et al. [12]	PIONEER 6	2019	69-week	Semaglutide 14 mg	1591	66 ± 7	31.9	14.7 ± 8.5	8.2 ± 1.6	91.0 ± 21.4
				Placebo	1592	66 ± 7	31.4	15.1 ± 8.5	8.2 ± 1.6	90.8 ± 21.0
Pieber et al. [27]	PIONEER 7	2019	52-week	Semaglutide	253	56.9 (9.7)	43	8.6 (6.3)	8.3 (0.6)	88.9 (19.6)
				Sitagliptin 100 mg	251	57.9 (10.1)	44	9.0 (6.2)	8.3 (0.6)	88.4 (20.1)
Zinman et al. [34]	PIONEER 8	2019	26/52-week	Semaglutide 3 mg	184	61 (9)	44.6	15.1 (7.9)	8.2 (0.7)	85.9 (21.5)
				Semaglutide 7 mg	182	60 (10)	43.3	16.2 (8.6)	8.2 (0.7)	87.1 (23.6)
				Semaglutide 14 mg	181	61 (10)	53.0	14.1 (8.0)	8.2 (0.7)	84.6 (21.0)
				Placebo	184	60 (10)	42.9	14.8 (7.9)	8.2 (0.7)	86.0 (21.4)
Lingvay et al. [23]	SUSTAIN 8	2019	52-week	Semaglutide 1.0 mg	394	55.7 (11.1)	43	7.5 (5.9)	8.3 (1.0)	90.6 (22.6)
				Canagliflozin300 mg	394	57.5 (10.7)	49	7.2 (5.4)	8.2 (1.0)	89.8 (22.6)
Zinman et al. [25]	SUSTAIN 9	2019	30-week	Semaglutide 1.0 mg	151	57.5 (8.9)	41.1	9.8 (6.3)	8.0 (0.8)	89.6 (19.5)
				Placebo	151	56.6 (10.1)	42.4	9.6 (5.9)	8.1 (0.8)	93.8 (22.3)
Capehorn et al. [15]	SUSTAIN10	2020	30-week	Semaglutide 1.0 mg	290	60.1 (10.5)	44.8	9.6 (6.1)	8.2 (0.9)	96.6 (21.0)
				Liraglutide 1.2 mg	287	58.9 (10.0)	41.8	8.9 (5.7)	8.3 (1.0)	97.2 (21.7)
Yamada et al. [24]	PIONEER 9	2020	52-week	Semaglutide 3 mg	49	58 (9)	27	7.4 (5.5)	8.1 (0.8)	71.4 (14.3)
				Semaglutide 7 mg	49	60 (10)	27	7.4 (5.6)	8.3 (1.0)	71.3 (10.8)
				Semaglutide 14 mg	48	61 (9)	17	7.9 (5.9)	8.0 (0.9)	68.0 (13.0)
				Placebo	49	59 (9)	18	8.4 (6.0)	8.3 (1.1)	70.3 (12.4)
				Liraglutide 0.9 mg	48	59 (10)	19	6.7 (5.2)	8.3 (0.8)	74.7 (15.4)
Yabe et al. [19]	PIONEER10	2020	52-week	Semaglutide 3 mg	131	59 (10)	24	9.4 (6.3)	8.2 (0.9)	71.5 (16.0)
				Semaglutide 7 mg	132	58 (11)	32	9.3 (6.3)	8.3 (0.9)	72.7 (16.4)
				Semaglutide 14 mg	130	57 (10)	23	9.1 (6.4)	8.4 (1.0)	72.6 (15.2)
				Dulaglutide 0.75 mg	65	61 (9)	22	9.9 (6.3)	8.4 (0.9)	71.2 (14.3)
Frías et al. [9]	SURPASS 2	2021	40-week	Semaglutide 1 mg	469	56.9 ± 10.8	52.0	8.3 ± 5.80	8.25 ± 1.01	93.7 ± 21.12
				Tirzepatide 5 mg	470	56.3 ± 10.0	56.4	9.1 ± 7.16	8.32 ± 1.08	92.5 ± 21.76
				Tirzepatide 10 mg	469	57.2 ± 10.5	49.3	8.4 ± 5.90	8.30 ± 1.02	94.8 ± 22.71
				Tirzepatide 15 mg	470	55.9 ± 10.4	54.5	8.7 ± 6.85	8.26 ± 1.00	93.8 ± 21.83
Davies et al. [11]	STEP 2	2021	68-week	Semaglutide 1.0 mg	403	56 (10)	50.4	7.7 (5.9)	8.1 (0.8)	99.0 (21.1)
				Semaglutide 2.4 mg	404	55 (11)	55.2	8.2 (6.2)	8.1 (0.8)	99.9 (22.5)
				Placebo	403	55 (11)	47.1	8.2 (6.2)	8.1 (0.8)	100.5 (20.9)
Ji et al. [18]	SUSTAIN China	2021	30-week	Semaglutide 0.5 mg	288	53.0 (11.4)	44.4	6.3 (5.4)	8.1 (0.9)	77.6 (16.4)
				Semaglutide 1.0 mg	290	53.0 (10.6)	46.9	6.7 (4.9)	8.1 (0.9)	76.1 (16.3)
				Sitagliptin 100 mg	290	53.1 (10.4)	36.2	6.1 (5.2)	8.1 (0.9)	75.5 (14.7)
Kellerer et al. [22]	SUSTAIN 11	2022	52-week	Semaglutide 1.0 mg/0.5 mg	874	60.8 (9.4)	49.1	13.4 (6.8)	8.6 (0.7)	87.6 (18.1)
				Insulin aspart	874	61.5 (9.5)	48.4	13.4 (6.5)	8.5 (0.7)	88.1 (18.4)
Gullaksen et al. [31]		2023	32-week	Semaglutide 1.0 mg	20	70.1 ± 6.8	15	8.5 (3.5–16.0)	7.5 (6.9–7.8)	94.9 (91.5, 98.4)
				Empagliflozin 10 mg	20	69.6 ± 6.0	35	10.0 (5.0–18.5)	7.4 (6.9–7.6)	94.9 (91.5, 98.4)
Takahashi et al. [30]	SWITCH-SEMA 1	2023	24-week	A: Semaglutide (0.25–1.0 mg)	19	59.6 (49.0–72.0)	36.8	NA	NA	NA
				B: Semaglutide (0.25–1.0 mg)	31	64.6 (54.0–75.0)	35.5	NA	NA	NA
				A: Liraglutide	18	60.9 (49.5–72.3)	33.3	NA	NA	NA
				B: Dulaglutide	32	60.8 (49.0–71.8)	59.4	NA	NA	NA

*HbA1c* glycated hemoglobin A1c, *ER* extended release, *OAD* oral antidiabetic drug, *NA* not available

### Quality assessment

Risk of bias (RoB) assessment was performed independently by 2 reviewers (W.W., Y.-S.L.) using the Cochrane Risk of Bias Assessment tool, and disagreements were resolved by discussion [8]. Selection bias, performance bias, detection bias, attrition bias, reporting bias, and other bias were graded as low, high or unclear risk.

### Statistical analyses

Statistical analyses were performed using Review Manager (RevMan) version 5.4 and Stata version 14. The mean changes in SBP, DBP, HbA1c and body weight from baseline were used as investigated parameters, expressed as WMDs and corresponding 95% CIs. The *I*^*2*^ index was used to assess the likelihood of statistical heterogeneity (an *I*-squared >50% is considered representative of important statistical heterogeneity). Depending on heterogeneity, the pooled outcomes were calculated using fixed- or random-effects models. When there was high heterogeneity, random-effects model was selected. Otherwise, fixed-effects model was used. Potential publication bias and related biases were evaluated by funnel plots, Begg’s funnel plot asymmetry and Egger’s test. In addition, meta-regression analysis and sensitivity analysis were performed in this meta-analysis.

## Results

### Literature search and study characteristics

**Fig. 1:**
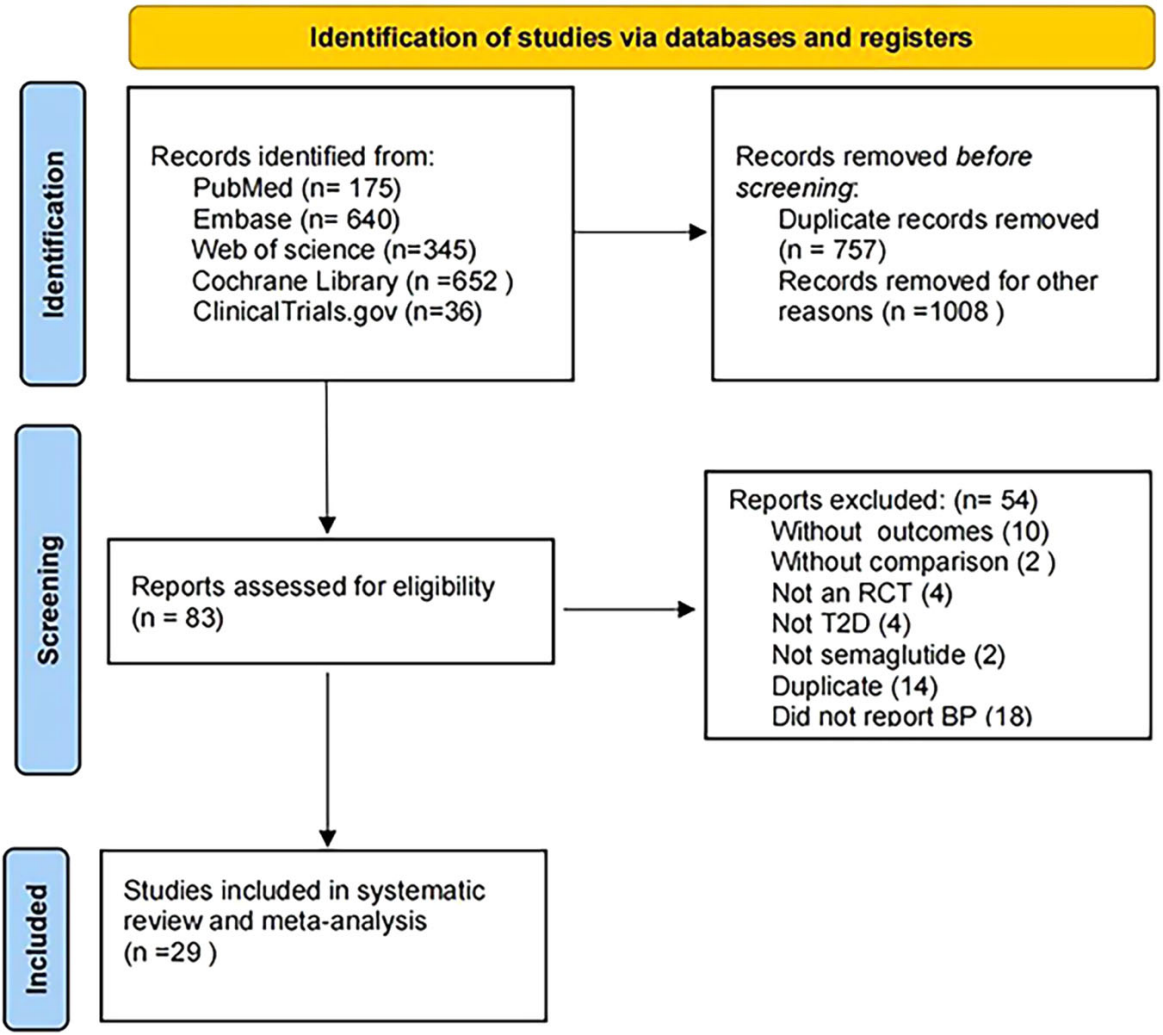
PRISMA (Preferred Reporting Items for Systematic Reviews and Meta-Analysis) flow chart of studies included in the meta-analysis. RCT randomized controlled trial, T2D type 2 diabetes, BP blood pressure

After initial database searches and manual screening of the literature, a total of 1848 unique citations were found, and 29 studies (*n* = 26985) published between 2016 and 2023 were eventually included. The flow chart of this review is displayed in Fig. 1. Ten trials (*n* = 9541) included oral semaglutide that was administered once daily, eighteen trials (*n* = 16814) included once-weekly subcutaneous injection of semaglutide, and one study (*n* = 630) evaluated both oral and subcutaneous semaglutide [9–37]. Doses of oral semaglutide ranged from 2.5 mg to 40 mg/day with sample sizes ranging from 48 to 1591 participants. The dosage of subcutaneous semaglutide ranged from 0.25 mg to 2.4 mg/day, and the sample sizes ranged from 19 to 874 participants. Detailed characteristics are shown in Table 1.

### Meta-analysis results

**Fig. 2:**
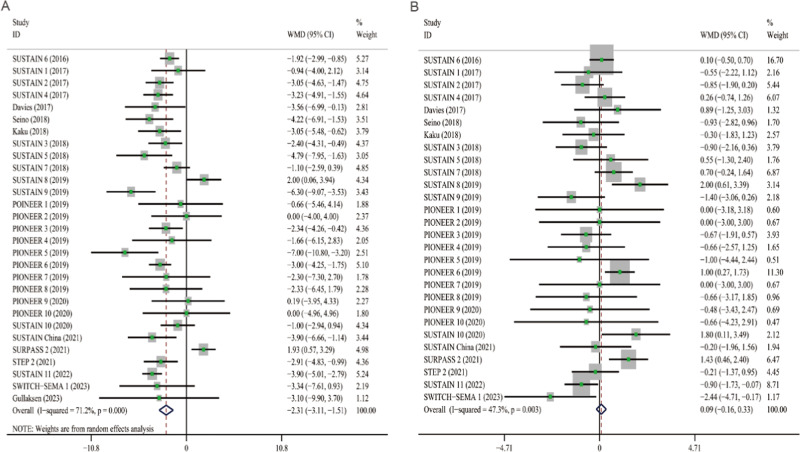
Forest plot of semaglutide vs. placebo or other antidiabetic drugs showing the pooled WMD for BP. **A** SBP (Random-effects model). **B** DBP (Fix-effects model). Each study is depicted by green squares (WMD) and widths (95% CI). The pooled WMD is presented by dark blue rhombuse and width (95% CI). WMD weighted mean difference, CI confidence interval, BP blood pressure, SBP systolic blood pressure, DBP diastolic blood pressure

**Fig. 3:**
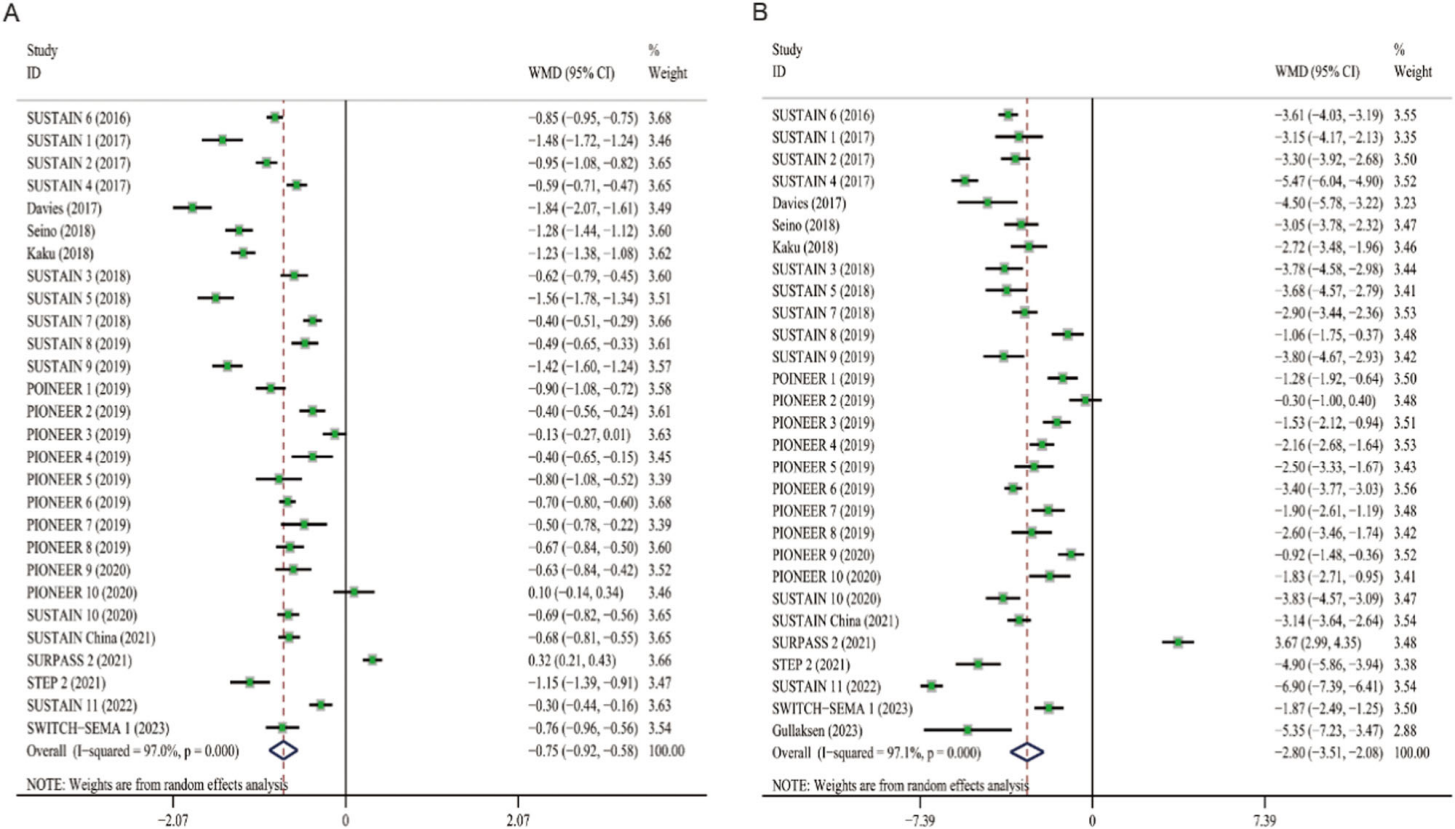
Forest plot of semaglutide vs. placebo or other antidiabetic drugs showing the pooled WMD for HbA1c and body weight (Random-effects model). **A** HbA1c. **B** Body weight. Each study is depicted by green squares (WMD) and widths (95% CI). The pooled WMD is presented by dark blue rhombuse and width (95% CI). WMD weighted mean difference, CI confidence interval, HbA1c glycated hemoglobin A1c

A mean reduction in SBP from baseline was indicated for semaglutide (WMD: −2.31, 95% CI: −3.11 to −1.51) across all trials. The trials had high heterogenetiy (*I*^*2*^ = 71.2%), so the random-effects model was selected for meta-analysis. The effect of semaglutide on SBP in individual studies ranged from −7.00 to 2.00 mmHg (Fig. 2A). The mean difference in DBP from baseline was 0.09 mmHg (95% CI: −0.16 to 0.33) across all studies, which was not statistically significant (Fig. 2B). The heterogeneity of this analysis was low (*I*^*2*^ = 47.3%), so the fixed-effects model was selected. Individual studies have shown therapeutic effects ranging from −2.44 to 2.00 mmHg. In addition, compared to placebo or other AHAs, semaglutide can reduce HbA1c by 0.75% (95% CI: −0.92 to −0.58) and weight by 2.80 kg (95% CI: −3.51 to −2.08), respectively (Fig. 3A, B).

### Subgroup analysis

**Fig. 4:**
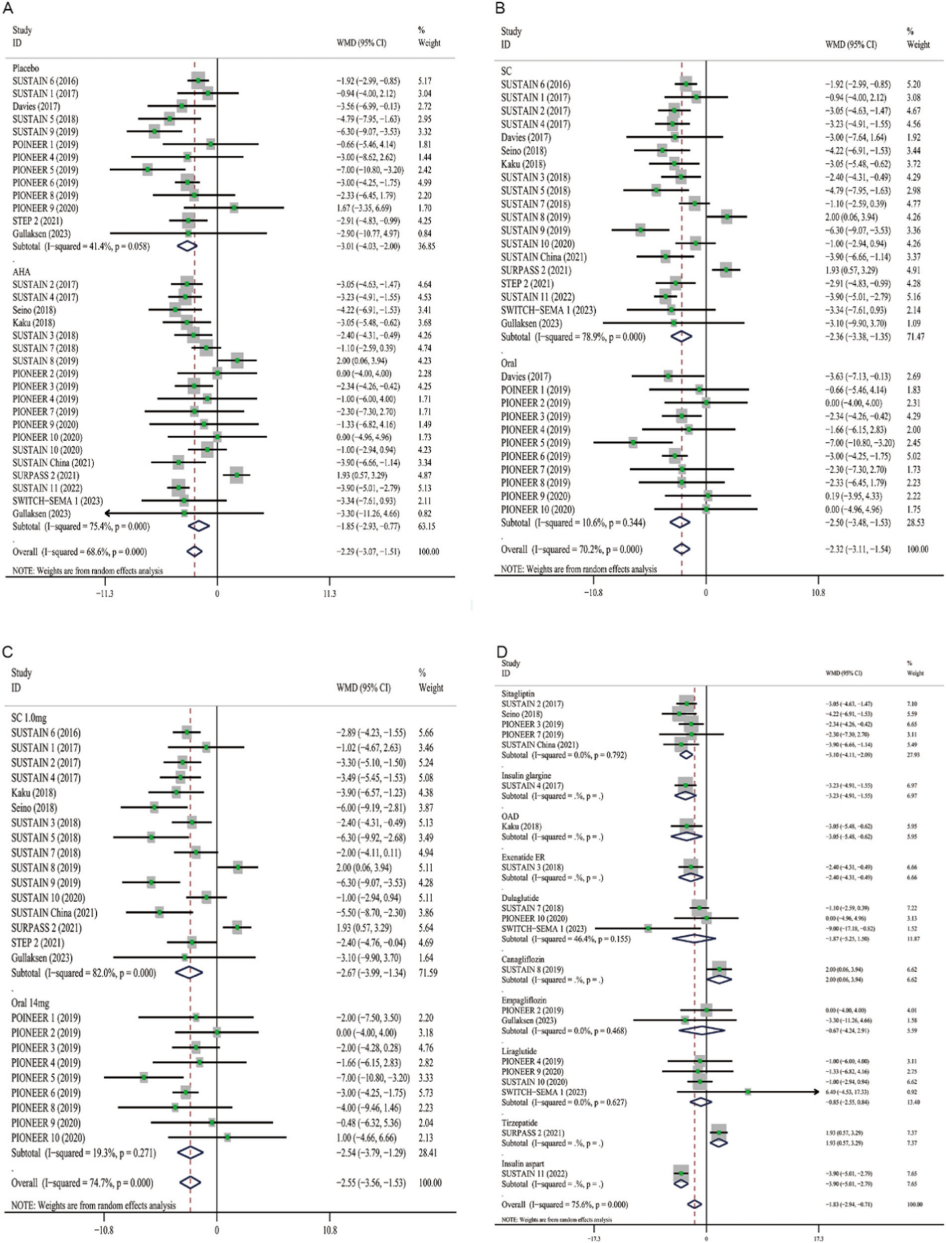
Forest plots of semaglutide regulate SBP. **A** Placebo and AHAs. **B** Subcutaneous and oral semaglutide. **C** Subcutaneous 1.0 mg and oral 14 mg semaglutide. **D** Different hypoglycemic drugs. Each study is depicted by green squares (WMD) and widths (95% CI). The pooled WMD is presented by dark blue rhombuse and width (95% CI). WMD weighted mean difference, CI confidence interval, AHAs antihyperglycemic agents, OAD oral antidiabetic drug, ER extended release

**Fig. 5:**
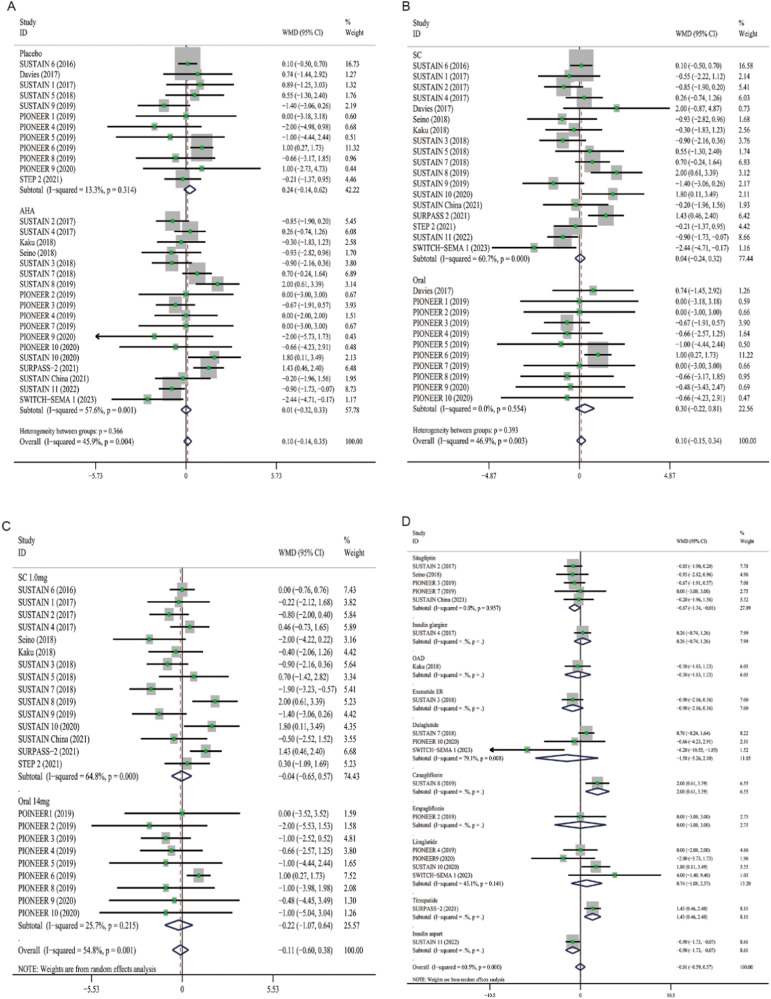
Forest plots of semaglutide regulate DBP. **A** Placebo and AHAs. **B** Subcutaneous and oral semaglutide. **C** Subcutaneous 1.0 mg and oral 14 mg semaglutide. **D** Different hypoglycemic drugs. Each study is depicted by green squares (WMD) and widths (95% CI). The pooled WMD is presented by dark blue rhombuse and width (95% CI). WMD weighted mean difference, CI confidence interval, AHAs antihyperglycemic agents, OAD oral antidiabetic drug, ER extended release

Semaglutide significantly reduced SBP levels (WMD: −3.01, 95% CI: −4.03 to −2.00) compared with placebo, and its effect was also superior to other AHAs (WMD: −1.85, 95% CI: −2.93 to −0.77) (Fig. 4A). Our analyses indicated that patients with oral semaglutide suggested a total reduction of −2.50 mmHg in SBP (95% CI: −3.48 to −1.53), patients with subcutaneous semaglutide showed an overall decrease of −2.36 mmHg (95% CI: −3.38 to −1.35) (Fig. 4B). Furthermore, there was no significant difference in terms of SBP reduction between 14 mg oral semaglutide (WMD, −2.54; 95% CI: −3.79 to −1.29) and 1.0 mg subcutaneous semaglutide (WMD, −2.67, 95% CI: −3.99 to −1.34) (Fig. 4C). When grouped by different antihyperglycemic agents, the combined WMD for insulin aspart was −3.90 mmHg (95% CI: −5.01 to −2.79), whereas it was 2.00 mmHg (95% CI: 0.06 to 3.94) for canagliflozin (Fig. 4D). The analysis showed that the antihypertensive effect of canagliflozin may be greater. With different control groups and administration routes, semaglutide did not significantly reduce DBP (Fig. 5).

### Meta-regression analyses

To investigate the high interstudy heterogeneity (*I*^*2*^ = 71.2%), we performed meta-regression analyses. The results of meta-regression indicated a significant association between SBP reduction differences and the variety of control groups (*P* < 0.05). The publication year and administration route of semaglutide were not significantly associated with heterogeneity (*P* > 0.05).

### Sensitivity analyses

We performed sensitivity analyses to estimate the stability of the results via sequentially excluding the results of each individual study. Sensitivity analyses showed that no single article had a strong influence on the results of the study.

### Risk of bias assessment

**Fig. 6:**
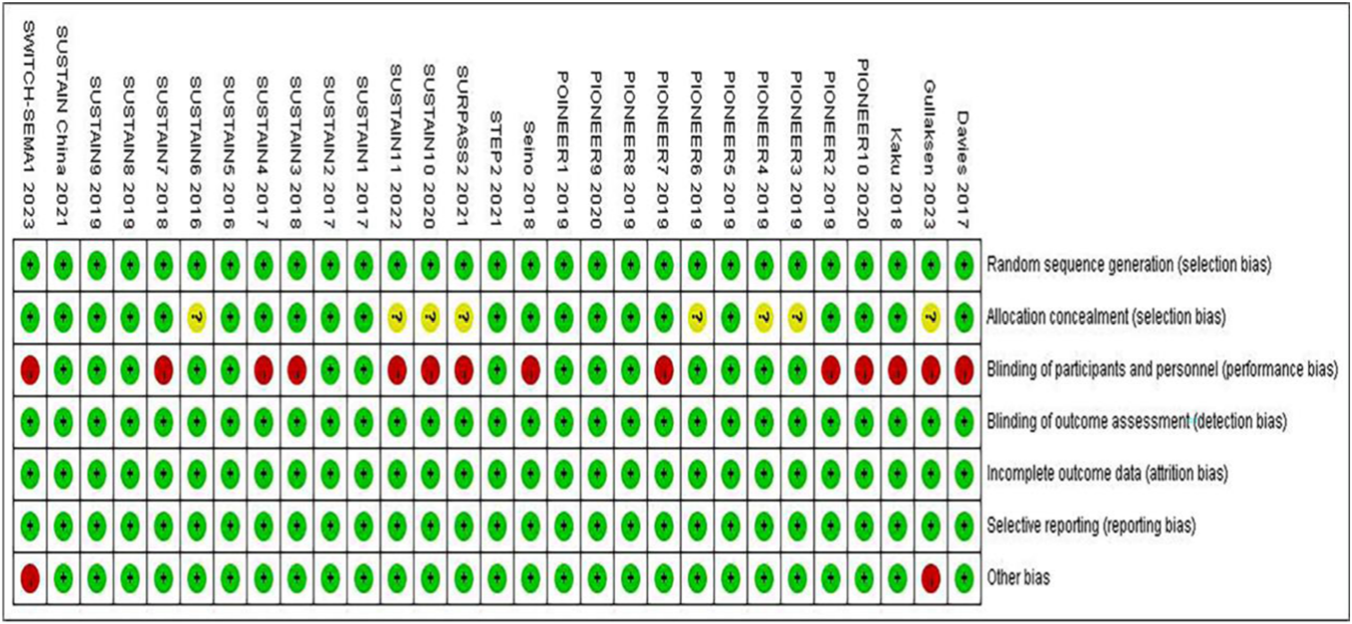
Risk of bias graph and summary for these 29 included studies. The green indicates a ‘low’ risk of bias; The yellow indicated an ‘unclear’ risk of bias; The red indicates a ‘high’ risk of bias

The risk of bias assessment for all included articles is presented in Fig. 6. Eight studies had an unclear risk of bias in allocation concealment. Fourteen trials were evaluated as having high performance bias because they used an open-label design. Two studies were assessed as having high other bias because of their small sample sizes.

### Publication bias

**Fig. 7:**
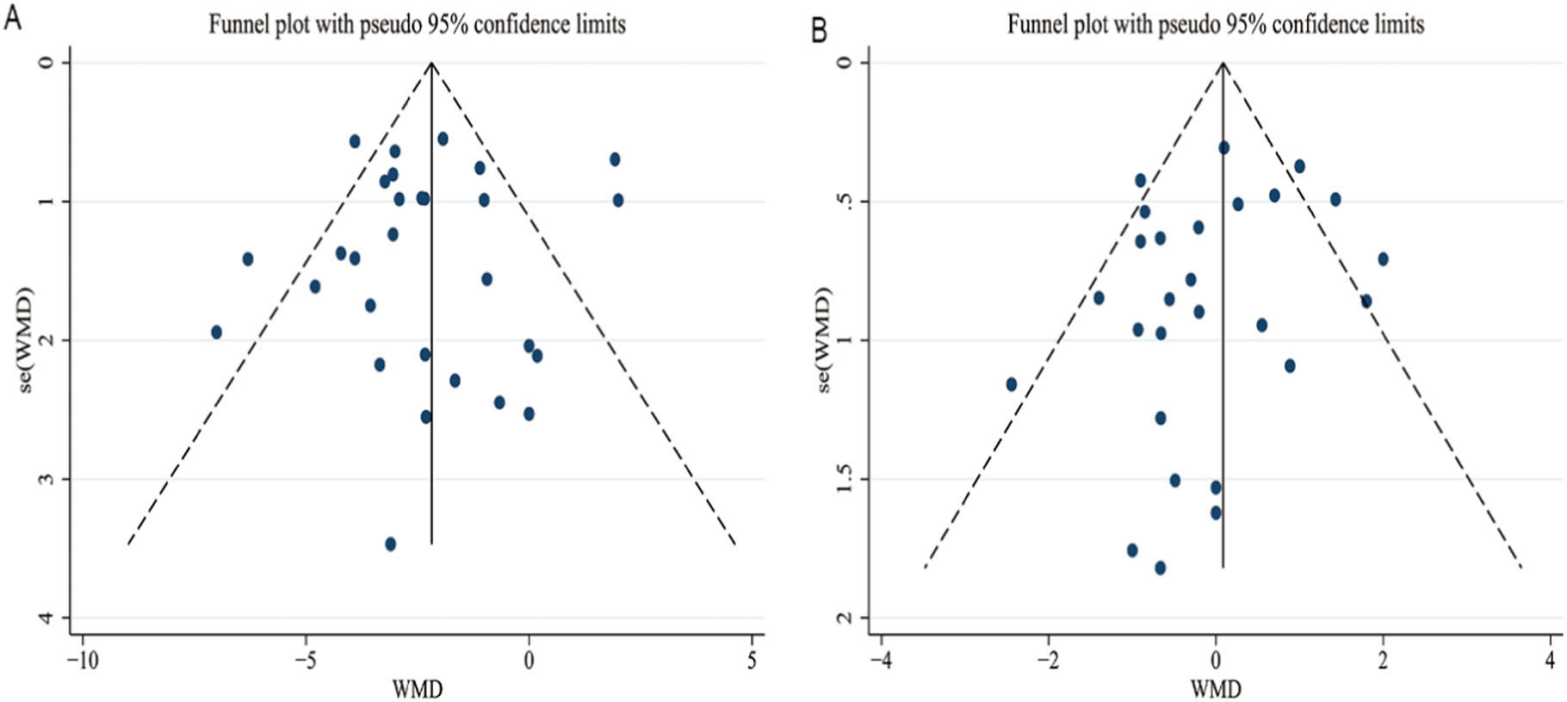
Funnel plots with publication bias for the analysis of the effects of semaglutide separately on SBP and DBP. **A** SBP. **B** DBP. The dark blue dots represent the results of each study. SBP systolic blood pressure, DBP diastolic blood pressure, WMD weighted mean difference, se standard error

Visual inspection of funnel plot symmetry did not indicate publication bias for SBP or DBP level analyses between semaglutide–treated groups and placebo/AHA-control groups (Fig. 7). Egger’s test and Begg’s test displayed that no publication bias was discovered in our meta-analysis of BP-lowering effects (*P* > 0.05 for all results).

## Discussion

This systematic review focuses on the regulatory effect of semaglutide on BP in subjects with T2DM. This meta-analysis of 29 RCTs involving 26985 individuals suggests that semaglutide reduces SBP by 2.31 mmHg compared with placebo or a variety of AHAs, including other GLP-1 RAs, oral hypoglycemic agents, basal insulin glargine, faster-acting insulin aspart, a dual GLP-1 RA and glucose-dependent insulinotropic tirzepatide. In terms of oral and subcutaneous formulations of semaglutide, changes in study design did not obviously alter the estimated therapeutic effect. In the subgroup analyses, subcutaneous semaglutide with the approved antihyperglycemic dose of 1.0 mg was not inferior to 14 mg of oral semaglutide, both with SBP reduction remaining at approximately 2 mmHg. Research has found that a reduction of only 2 mmHg in SBP can reduce the incidence of cardiovascular events and mortality risk [38].

A recent meta-analysis including 6 trials and 4744 participants indicated that semaglutide reduced DBP by 2.5 mmHg in obese patients without DM [39]. However, in this review, there was no statistically significant difference in the effect of semaglutide on DBP compared with the control groups. The effect of semaglutide on BP in this paper was lower than expected, which may be related to the inclusion of low-dose semaglutide trials in the study. In addition, the antihypertensive effects of some hypoglycemic agents were greater than that of semaglutide, which may also be an influencing factor.

People with diabetes are often accompanied by hypertension, obesity, dyslipidemia, fatty liver and hyperuricemia [40]. These are important risk factors for CVD and related complications, which need to be considered in diabetes management. GLP-1 RAs have a variety of positive effects on individuals with diabetes, including improving glycaemic control, reducing body weight and BP, ameliorating lipid profiles, decreasing oxidative stress and inflammatory markers, advancing renal outcomes and improving subclinical atherosclerosis and endothelial dysfunction, thereby reducing and possibly preventing cardiovascular events [41].

This meta-analysis mainly included the SUSTAIN program, which involved weekly subcutaneous injections of semaglutide, and the PIONEER program, which involved daily oral administration of semaglutide. Among them, SUSTAIN-6 and PIONEER-6 were the cardiovascular outcome trials (CVOTs) that studied the primary adverse cardiovascular outcomes (nonfatal stroke, nonfatal myocardial infarction, or death from cardiovascular causes). The two trails had similar hazard ratios, which may indicate that the cardiovascular effects of semaglutide are unrelated to the drug formulation.

Semaglutide showed CV benefits in PIONEER-6 and SUSTAIN-6, which are consistent with other CVOTs studying GLP-1 RAs, such as EXSCEL, HARMONY, LEADER, REWIND and ELIXA, which included patients on exenatide, albiglutide, liraglutide, dulaglutide and lixisenatide, respectively [41, 42]. In addition, research has shown that the administration of liraglutide or exenatide during primary angioplasty for acute myocardial infarction can effectively reduce the infarct size without affecting left ventricular function [43]. As a result, the status of GLP-1 RAs in the T2D treatment pathway has been significantly improved. In patients with T2D combined with arteriosclerotic cardiovascular disease (ASCVD) or very high cardiovascular risk, GLP-1 RAs have been recommended as one of the preferred combined medications.

The use of semaglutide has made a breakthrough in reducing cardiovascular risk in patients with T2D, although it can lead to an increase in pulse rate, which seems to have nothing to do with adverse cardiac events. Furthermore, we should be aware that when applying semaglutide in older individuals with long-term diabetes, in addition to causing gastrointestinal adverse reactions (mild or moderate nausea, vomiting, constipation, diarrhea), it may also increase the risk of diabetic retinopathy complications, which is still a matter of debate [10]. The FOCUS trial (NCT03811561), which is being conducted to study the long-term effects of semaglutide in diabetic eye disease, is ongoing and is expected to end in 2027.

Ferdinand et al. found that one-third of the effect of dulaglutide on reducing SBP depends on weight loss [38]. We also investigated the changes in HbA1c and body weight after treatment with semaglutide, and found that HbA1c decreased by 0.75% and body weight reduced by 2.80 kg, indicating that weight loss and SBP reduction were synchronized. Therefore, we can speculate that semaglutide reduces SBP by decreasing body weight. However, the extent to which weight loss leads to a decrease in blood pressure requires further investigation in the future.

Recent studies have found that the BP reduction seen with GLP-1 RAs is not related to blood glucose regulation [44]. Its antihypertensive mechanism may involve improving endothelial function, modulating smooth muscle cell phenotype, activating GLP-1R in the brainstem, weight loss, and natriuretic effects [44, 45]. Chiriacò et al. found that the dysregulation of BP circadian rhythm can increase subclinical organ damage and mortality [46]. The glucose-lowering drug pioglitazone and the sodium glucose cotransporter-2 inhibitor may be able to counteract the changes in the circadian rhythm of BP. However, it is unclear whether semaglutide, in addition to its known antihypertensive effect, can also improve the dysregulation of BP circadian rhythm. Future research should strengthen the direct exploration of semaglutide antihypertensive mechanisms.

The DIRECT trial shows that weight loss can significantly mitigate the progression of diabetes in obese patients with T2D [47]. Some antihyperglycemic agents used to treat T2D, such as insulin, sulfonylureas and thiazolidinediones, can lead to weight gain and frequent episodes of hypoglycemia, which are associated with reduced quality of life and increased cardiovascular events. Therefore, if the use of hypoglycemic drugs such as semaglutide, not only reduces body weight but also carries a low risk of hypoglycemia, it seems eminently sensible that semaglutide can be used as an alternative to antihypertensive treatment for hypertensive diabetes patients. Furthermore, once-weekly subcutaneous semaglutide injections reduce the patient’s injection burden, and once-daily oral administration of semaglutide overcomes the barriers of injectable therapies and makes medication more convenient for T2D. Thus, semaglutide can improve treatment satisfaction, reduce anxiety, and increase patient compliance.

In addition to the performance bias in some RCTs using the open-label design, this study has high-quality research methods and a low risk of bias. We performed meta-regression and subgroup analysis to search for possible sources of heterogeneity and found that differences in the control groups may be associated with heterogeneity.

This review has several potential limitations. First, many of the included studies did not provide the mean BP at baseline, and it was not clear whether the patients were comorbid with hypertension or whether they were co-administered with antihypertensive medications. Because the only BP data available were for baseline changes, the meta-analysis may also be prone to bias. Second, BP was not the primary endpoint in most of the included studies, so all reported BP data must be treated with caution. Next, participants in this review did not have uniform hypoglycemic backgrounds, which may also contribute to heterogeneity in our results. Once again, this analysis did not compare the difference in whether semaglutide has an antihypertensive effect among different races. Finally, the duration of the included studies was sufficiently long to evaluate the primary outcome. However, because the follow-up time was generally short, it is still necessary to investigate the long-term antihypertensive effect of semaglutide.

## Conclusions

Our analysis suggests that semaglutide, either oral or subcutaneous, can significantly reduce SBP in subjects with T2D. For diabetes patients with hypertension, the antihypertensive effect of semaglutide may be greater than the 2.31 mmHg in this paper, but the real-world effect has yet to be determined. Future studies to uncover the underlying BP-lowering mechanisms of semaglutide will benefit more individuals with diabetes by increasing the understanding these associations.

### Supplementary information

Supplementary Information

## Data Availability

The data of this article are available from the corresponding author.
